# Development of fluorescent lateral flow immunoassay for SARS-CoV-2-specific IgM and IgG based on aggregation-induced emission carbon dots

**DOI:** 10.3389/fbioe.2022.1042926

**Published:** 2022-10-13

**Authors:** Jian Ju, Xinyu Zhang, Lin Li, Sagar Regmi, Guoqiang Yang, Shixing Tang

**Affiliations:** ^1^ Wenzhou Institute, University of Chinese Academy of Sciences, Wenzhou, China; ^2^ Oujiang Lab, Wenzhou, China; ^3^ School of Ophthalmology and Optometry, Wenzhou Medical University, Wenzhou, China; ^4^ Department of Pharmacology, School of Medicine, Case Western Reserve University, Cleveland, OH, United States; ^5^ Key Laboratory of Photochemistry, Institute of Chemistry, University of Chinese Academy of Sciences, Chinese Academy of Sciences, Beijing, China; ^6^ Department of Epidemiology, School of Public Health, Southern Medical University, Guangzhou, China

**Keywords:** aggregation-induced emission carbon dots, COVID-19, SARS-CoV-2 antibody detection, lateral flow assay, internal standard calibration

## Abstract

Understanding the dynamic changes in antibodies against severe acute respiratory syndrome coronavirus 2 (SARS-CoV-2) is essential for evaluating the effectiveness of the vaccine and the stage for the recovery of the COVID-19 disease. A rapid and accurate method for the detection of SARS-CoV-2-specific antibodies is still urgently needed. Here, we developed a novel fluorescent lateral flow immunoassay (LFA) platform for the detection of SARS-CoV-2-specific IgM and IgG by the aggregation-induced emission carbon dots conjugated with the SARS-CoV-2 spike protein (SSP). The aggregation-induced emission carbon dots (AIE-CDs) are one of the best prospect fluorescent probe materials for exhibiting high emission efficiency in both aggregate and solid states. The AIE-CDs were synthesized and displayed dual fluorescence emission, which provides a new perspective for the design of a high sensitivity testing system. In this work, the novel LFA platform adopted the AIE carbon dots, which are used to detect SARS-CoV-2-specific IgM and IgG conveniently. Furthermore, this sensor had a low LOD of 100 pg/ml. Therefore, this newly developed strategy has potential applications in the areas of public health for the advancement of clinical research.

## Introduction

The coronavirus disease 2019 (COVID-19), caused by severe acute respiratory syndrome coronavirus 2 (SARS-CoV-2), is still spreading, posing a huge threat to human health (Ienca and Vayena, 2020; Regmi et al., 2020; Tuttle, 2020). As the viral infection progresses to the lower trachea and even to the lungs, the disease rapidly develops into a severe infection which is characterized by acute respiratory distress syndrome (ARDS), and the condition of patients with severe illness may deteriorate to organ dysfunction and even to death (Hou et al., 2020; Kang et al., 2022). It is a tragic milestone that more than one million have died in 2022 from COVID-19 around the globe, and thousands of people are struggling for their lives in hospitals. To prevent the spread of this COVID-19 and control the pandemic, it is very urgent to develop a rapid and accurate method for detecting SARS-CoV-2 in the diagnosis and clinical treatment.

According to the guideline for the COVID-19 diagnosis and treatment (Xu et al., 2020), the virus nucleic acid quantitative reverse transcription polymerase chain reaction (qRT-PCR) is the standard method for the clinical diagnosis of SARS-CoV-2 because of its mature technique and high sensitivity (Liang et al., 2022). However, there are still some unfavorable factors that could not be overcome, such as tests that require certified laboratories, expensive equipment, and time-consuming processes (Kanji et al., 2021). In addition, false-negative results were unavoidable due to instability of the reagents, virus load, and unskilled operation by technicians (Arevalo-Rodriguez et al., 2020; Lan et al., 2020). More importantly, as a sophisticated diagnostic technology, all the relative reagents are mandated to be transported and stored in the cold-chain logistics, which limits the application in point-of-care testing (POCT) (Huang et al., 2020). The immuno-based assays which depend on high affinity between the antigen and antibody, enzyme-linked immunosorbent assay (ELISA) (Gillot et al., 2020), chemiluminescence immunoassay (CLIA) (Padoan et al., 2020), and lateral flow assay (LFA) (Zhang et al., 2021) methods are widely used in the SARS-CoV-2 antibody/antigen test. In particular, the LFA technique is more acceptable in the large-scale self-inspection process by the people due to its convenient operation, low cost, and independent of medical expertise (Posthuma-Trumpie et al., 2009; Alhabbab et al., 2022).

The SARS CoV-2 blood-specific antibody test is a rapid and specific method for diagnosing COVID-19. Immunoglobulin M (IgM) and Immunoglobulin G (IgG) were detectable in 94.1% and 100% of patients, respectively within 19 days after the symptom onset (Long et al., 2020). It was proposed that IgM generally appears in serum before IgG as the first line of immune defense against microbial infection (Long et al., 2020; Sun et al., 2020). The detection of IgM and IgG is suitable for the detection of a huge number of suspected cases and asymptomatic infections. In certain cases, the test effectively checks for the presence of anti-SARS-CoV-2 antibodies developed either in response to a COVID-19 infection or to a vaccine (Sun et al., 2020; Du et al., 2022). In countries with high vaccination rates, the application of antibody testing in the diagnosis of COVID-19 infection is significantly declining. However, according to the World Health Organization (WHO) statistics, one-third of the world’s population remains unvaccinated against COVID-19, including two-thirds of healthcare workers and three-quarters of older people in low-income countries. Recently, the WHO’s COVID-19 Technology Access Pool (C-TAP) has tried to accelerate the manufacture and sale of the COVID-19 serological antibody technology around the globe, so many people in more countries can have easier access to affordable diagnostics. Therefore, it is very important to establish a sensitive, selective, and low-cost detection method for SARS-CoV-2 antibodies.

At present, many types of signal markers have been identified, such as colorimetrically labeled gold dyes (Huang et al., 2020) and organic dye (Sato et al., 2022) and non-colorimetrically labeled fluorescent dyes (Zhang et al., 2020), quantum dots (Luo et al., 2022), magnetic beads (Liu et al., 2015), and microspheres (Yang et al., 2020). The fluorescence immunoassay received great attention due to its selectivity, sensitivity, and easy operation. Different from the conventional colloidal gold test strips on the market, fluorescent test strips can not only achieve qualitative detection but also quantitative detection (Liu and Rusling, 2021). The currently used commercial fluorescent probes are partly based on fluorescent proteins or complex chemical backbones, which are expensive to synthesize, express, and purify (Ji et al., 2020). Among the aforementioned fluorescent probes, carbon dots exhibit easy functionalization, hydrophilicity, luminescent stability, and biocompatibility properties (Gong et al., 2019; Liu et al., 2020; Ghaffarkhah et al., 2022). The carbon dot-based material is considered to be promising and competitive label probe candidates. However, few reports have used carbon dots alone as LFA applications due to the weak fluorescence intensity of single carbon dots (Wang et al., 2022a). Many researchers have utilized silicon spheres as matrix to obtain the stable luminescent composites (Wang et al., 2022a). Unfortunately, the integration of carbon dots into the nanosphere process usually requires precise control of the reaction conditions to avoid the aggregation of carbon dots, which may affect their fluorescence properties due to aggregation-caused quenching (ACQ) (Xu et al., 2019). In addition, the process of assembling carbon dots into the silicon spheres is time-consuming and increases the cost of the preparation. The aggregation-induced emission (AIE) materials can effectively avoid the phenomenon of ACQ which appear in most fluorescent materials. Although the synthesis steps (Kyarikwal et al., 2022) and optical signal regulation (Wang et al., 2022b) are complex, they are still attractive and have broad prospects in practical applications, such as industrial pollutant detection (Paul et al., 2022) and anti-counterfeiting ink application (Yang et al., 2019). Thus, the development of novel carbon dots with new optical properties that can be directly used as tags would be significant for the lateral flow test strip. In this study, aggregation-induced emission carbon dots (AIE-CDs) are prepared using a rapid one-pot hydrothermal method. The prepared AIE-carbon dots have typical AIE properties with dual emission (blue and red fluorescence) (Yang et al., 2019). More interestingly, the AIE-CDs tend to exhibit strong red emission at high concentration, which provides the new potential application for the concentrated solution. Moreover, we first constructed a novel LFA technology for the SARS-CoV-2-specific IgM and IgG by AIE-CDs used as fluorescence labels. A linear relationship between the ratio of the test line and the control line in the conventional fluorescence immunoassay and the concentration of SARS-CoV-2-specific IgM and IgG was identified. As expected, the detection limit of the assay was down to 100 pg/ml. Meanwhile, the ratio of the dual emission under the same excitation was also used for a stable internal standard. The determination results suggested that the method was sensitive, selective, simple, and convenient compared with the prior methods reported in the literature.

## Experimental

### Materials, reagents, and instrumentation

Thiourea, citric acid, and sucrose were provided by Shanghai Aladdin Biochemical Technology Co. Ltd. (Shanghai, China). N-(3-Dimethylaminopropyl)-N′-ethyl-carbodiimide hydrochloride (EDC) and polyethylene glycol (PEG, 600,000) were purchased from Sigma, China. N-Hydroxy succinimide (NHS) was bought from Bide Pharmatech Ltd (Shanghai, China). Bovine albumin (BSA) and trehalose were provided by Energy Chemical (Shanghai, China). Tween-20 was obtained from Beyotime (Shanghai, China). The SARS-CoV-2 S protein (73 KDa) was purchased from Genscript (Nanjing, China). Chicken-IgY (188 KDa) and goat anti-chicken IgY were purchased from Jiangsu Fanbo Biological Products Co., Ltd (Nanjing, China). The SARS-CoV-2 spike antibody IgM, IgG, mouse antihuman IgM antibody, and mouse antihuman IgG antibody were purchased from Fantibody (Chongqing, China). The nitrocellulose (NC) membrane was purchased from Whatman (Maidstone, United Kingdom), and ultrafiltration centrifuge tubes (50 K and 100 K) were obtained from Merck Millipore (Massachusetts, United States). The LFA materials like the plastic backing card, absorbent pad, glass fiber conjugate pad, and sample loading pad were provided by Jieyi Biotechnology (Shanghai, China).

The transmission electron microscopy (TEM) images of nanocomposites (including AIE-CDs and AIE-CD-SSP) were obtained on an FEI Talos F200S (Hillsboro, United States). The zeta potentials and dynamic light scattering (DLS) results were investigated using a Nano ZS ZEN3600 Zetasizer (Malvern, United Kingdom). The pictures of the AIE-CD-SSP LFA technology were detected using an inverted fluorescence microscope Axio Vert.A1 (Zeiss, Jena, Germany), with the blue light irradiation excitation wavelength at 420–485 nm and green light irradiation excitation wavelength at 460 nm–550 nm. The fluorescence signals of the AIE-CDs and AIE-CD-SSP were acquired using a fluoro-spectrophotometer (Hemai, Suzhou China). The UV absorption spectrum was obtained using a dual beam UV-vis spectrophotometer TU-19 PERSEE (Beijing, China). The BioTek Synergy H1 microplate reader (Vermont, United States) was used to test the fluorescence intensity.

### Preparation of AIE-CDs

In this experiment, AIE-CDs were synthesized by the hydrothermal method. The 3:1 mass ratio of thiourea and citric acid was dissolved in DMF (10 ml) and heated to 160°C in a 25-ml hydrothermal synthesis reactor for 6 h. After cooling to room temperature, the products were centrifuged at 10,000 rpm for 10 min. To obtain stable red-light AIE-CDs, the centrifuged supernatant of 5 ml was added dropwise into 40 ml NaOH (50 mg/ml) solution. The obtained AIE-CDs were stored in a refrigerator at 4°C for later use.

### Preparation of SARS-CoV-2 spike protein-conjugated AIE-CD-SSP

The AIE-CDs were conjugated with the labeled antibody of SARS-CoV-2 spike protein (SSP) through the classical carbodiimide coupling reaction. First, the surface carboxyl groups of AIE-CDs were activated. Then, 50 μl 0.1 M EDC was added to 1.5 ml of AIE-CDs (60 mg/ml) and incubated for 10 min at room temperature. Subsequently, 50 μl of 0.1 M NHS solution was poured into the aforementioned mixture to activate carbon dots; then, the pH was adjusted to 9 with glacial acetic acid (99.5%). After 1 h, SSP (20 µg) was added and incubated at 37°C for 3 h. Then, 50 μl 10% BSA was added to block the nonspecific adsorption sites of the AIE-CDs and continued incubation at 37°C for 1 h. The preparation process of the test line marker is the same as the aforementioned steps. AIE-CD-coated chicken IgY was named as AIE-CD-IgY. The mixture of the fluorescent complex was centrifuged at 3,500 rcf for 5 min at 4°C through an ultrafiltration centrifuge tube, and AIE-CD-SSP and AIE-CD-IgY were filtered using 50-K and 100-K ultrafiltration centrifuge membranes, respectively. After recovery of the liquid in the inner tube of the ultrafiltration tube, the AIE-CD signal probe was resuspended with a volume ratio of 1:2 re-solution buffer and stored at 4°C for future use.

### Fabrication of the LFA strip for the simultaneous detection of anti-SARS-CoV-2 IgM and IgG

**FIGURE 1:**
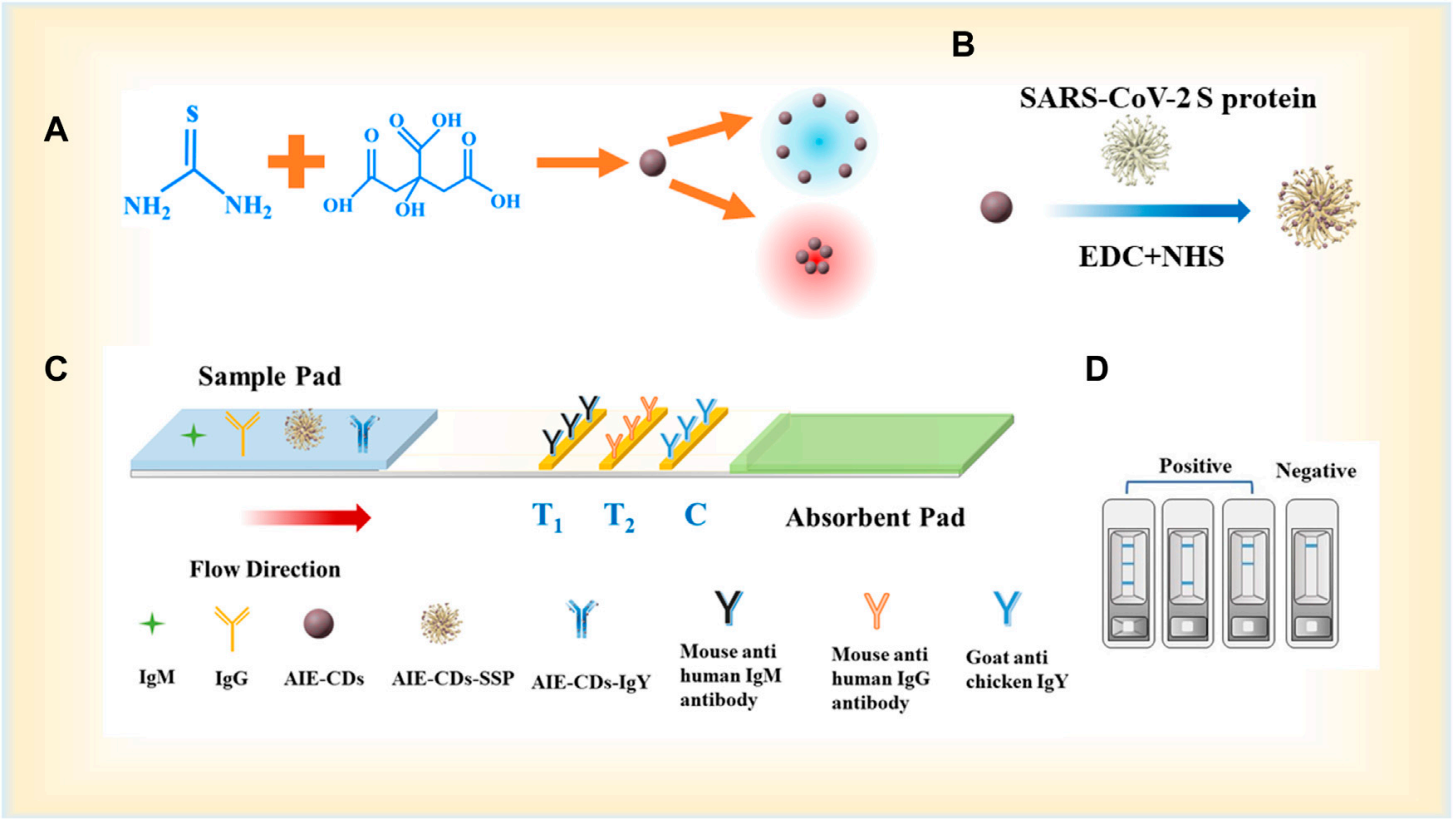
**(A)** Synthesis process of AIE-CDs; **(B)** synthesis process of fluorescent marker AIE-CD-SSP; **(C)** principle of the AIE-CD-SSP-based LFA strip for the detection of SARS-CoV-2 IgM/IgG; and **(D)** schematic diagram of test strip detection in positive and negative situations.

The synthesis of the AIE-CD fluorescent marker and the experimental principle of the AIE-CD-SSP-based LFA strip for the detection of anti-SARS-CoV-2 IgM and IgG are illustrated in Figure 1. The test strip was composed of three parts from left to right, namely, the sample pad, the NC membrane, and the absorption pad. The NC membrane was coated with the secondary antibody that was regarded as the detection area which has two test lines (T-line) and a control line (C-line). We used 0.5 mg/ml of mouse anti-human IgM and mouse anti-human IgG which were immobilized on the NC membrane to construct the test line (T-line), and 0.8 mg/ml of goat anti-chicken IgY was coated on the control line (C-line), with each test strip dispensed in 1 μl/cm. The fluorescent complex mixture of 4 μl AIE-CD-SSP and 1 μl AIE-CD-IgY and each test strip was used to detect SARS-CoV-2 IgM and IgG in the strip. When a human serum specimen containing SARS-CoV-2 IgM and IgG antibodies was dropped onto the sample pad, the specimen began to migrate toward the absorbent pad through capillary forces. Afterward, the membranes and binding pads were dried at a constant temperature of 37°C for 24 h and then transferred to a desiccator for storage. Then, the sample pad, NC film, and absorbent pad were glued to the bottom plate in sequence and then cut into widths of 3 mm for each strip.

### Performance of LFA for the detection of anti-SARS-CoV-2 S IgM and IgG

In this experiment, a 96-well plate was used to test the linear relationship of the intensity of the fluorescence detection. First, the mouse antihuman IgG, mouse antihuman IgM, and goat anti-chicken IgY were diluted to 0.5 mg/ml, 0.5 mg/ml, and 0.8 mg/ml, respectively, by adding 0.1 M NaCO_3_ containing 3% trehalose buffer. Then, approximately 100 μl coated mixed fluid, which includes 20 μl of the diluted antibody solution and 80 μl of 5% BSA coated on it, was taken in the well plate and incubated at 4°C overnight.

The mouse anti-human IgG and mouse anti-human IgM were taken in 21 (3 × 7) wells as test groups, and goat anti-chicken IgY was taken in 7 (1 × 7) wells as the control group. Then, approximately 70 μl of a concentration gradient of SARS-CoV-2 IgM and IgG was added to the well plate, followed by the addition of 10 μl AIE-CD-SSP. Then, the 96-well plate was incubated at 37°C for another 3 h. Finally, the liquid mixture in each well was removed and supplemented with 0.1M PBS. Then, the fluorescence intensity of each well was tested three times using the fluorescence microplate reader, and the linear regression calculation was recorded.

## Results and discussion

### Characterization of the AIE-CDs and fluorescent complexes

**FIGURE 2:**
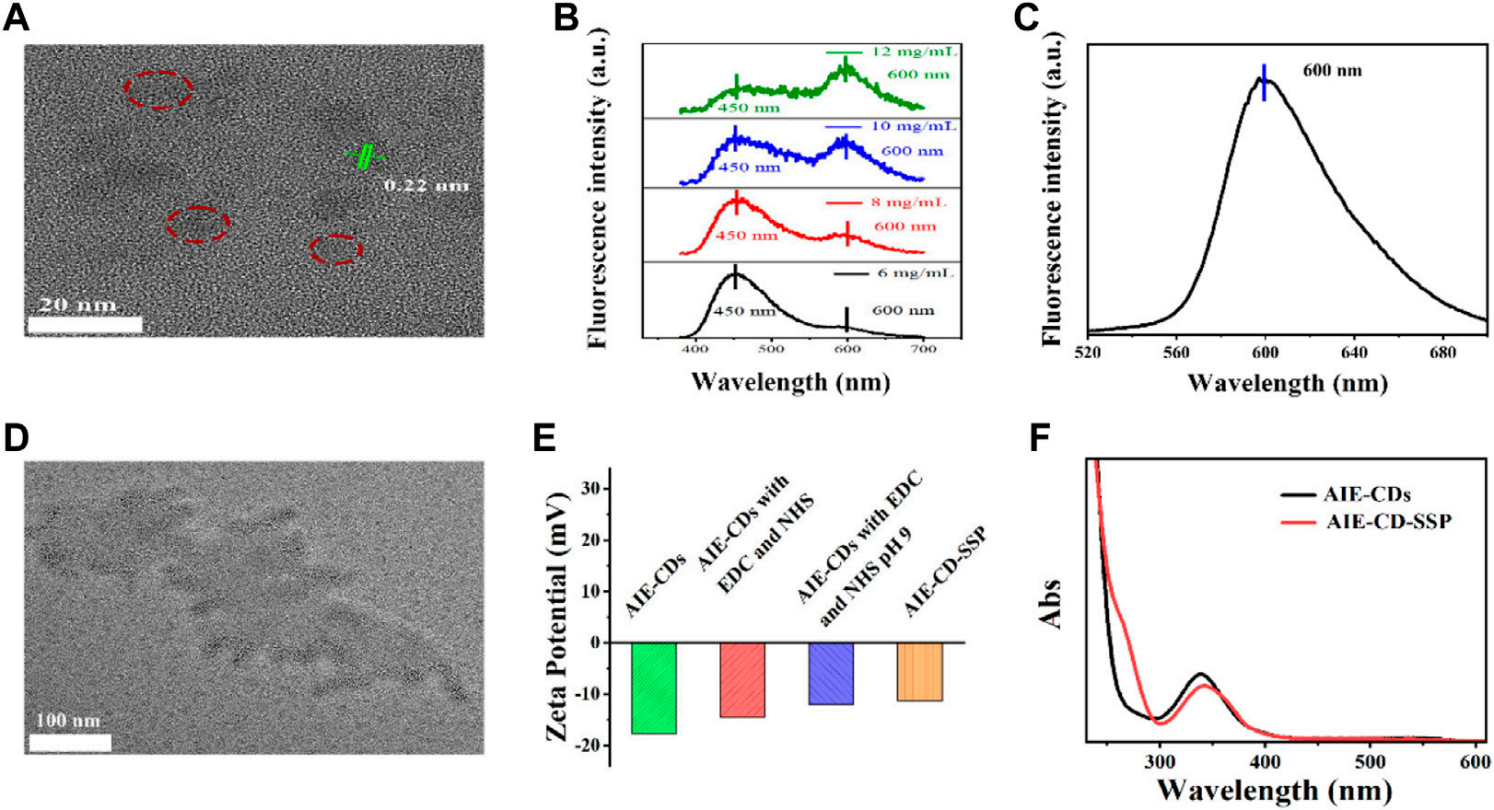
**(A)** Transmission electron microscope (TEM) image and the lattice pattern of AIE-CDs. **(B)** **(C)** Fluorescence spectrogram of original AIE-CDs under the excitation wavelength at 365 nm and 500 nm. **(D)** TEM image of AIE-CD-SSP. **(E)** Zeta potential of products at various stages prepared from AIE-CDs to AIE-CD-SSP. **(F)** UV spectra of AIE-CDs and AIE-CD-SSP.

The morphology of the AIE-CDs and AIE-CD-SSP was characterized by transmission electron microscopy (TEM). As shown in Figure 2A, AIE-CDs have good dispersibility; the average size is about 8 nm, and the lattice pattern is 0.22 nm. The fluorescence spectrogram of original AIE-CDs under excitation wavelengths at 365 nm and 500 nm is shown in Figures 2B,C, respectively. At the excitation wavelength of 365 nm, the fluorescence spectrogram showed two emission peaks at 450 nm and 600 nm. As the concentration gradually increased, the fluorescence intensity at 450 nm gradually weakened, while the red emissions at 600 nm gradually increased slightly, and this phenomenon was attributed to the aggregation-induced double emission of AIE-CDs. Also, at an excitation wavelength of 500 nm, the emission peak appeared at 600 nm. As shown in Figure 2D, AIE-CDs are uniformly dispersed on the SSP surface. The DLS results showed that the particle size of the AIE-CDs was 10 nm, which was larger than the TEM image, attributed to the good hydrophilicity of the AIE-CDs. The zeta potential was used to demonstrate that the final fluorescent complex was obtained. As shown in Figure 2E, the zeta potential of AIE-CDs was −17.7 mV, and the zeta potential of AIE-CDs after EDC and NHS activation was introduced as −14.5 mV, whereas that of AIE-CD-SSP substantially increased to −11.3 mV after SSP coupled with AIE-CDs. This result confirmed that SSP and AIE-CDs were successfully conjugated. The same experimental conclusion was verified in the UV-vis absorption spectrum. As shown in Figure 2F, compared with the original AIE-CDs, a new peak appeared at 263 nm corresponding to the characteristic peak of SSP, while the characteristic peak at 338 nm of AIE-CDs was red-shifted to 342 nm, which was due to the coupling of the protein.

**FIGURE 3:**
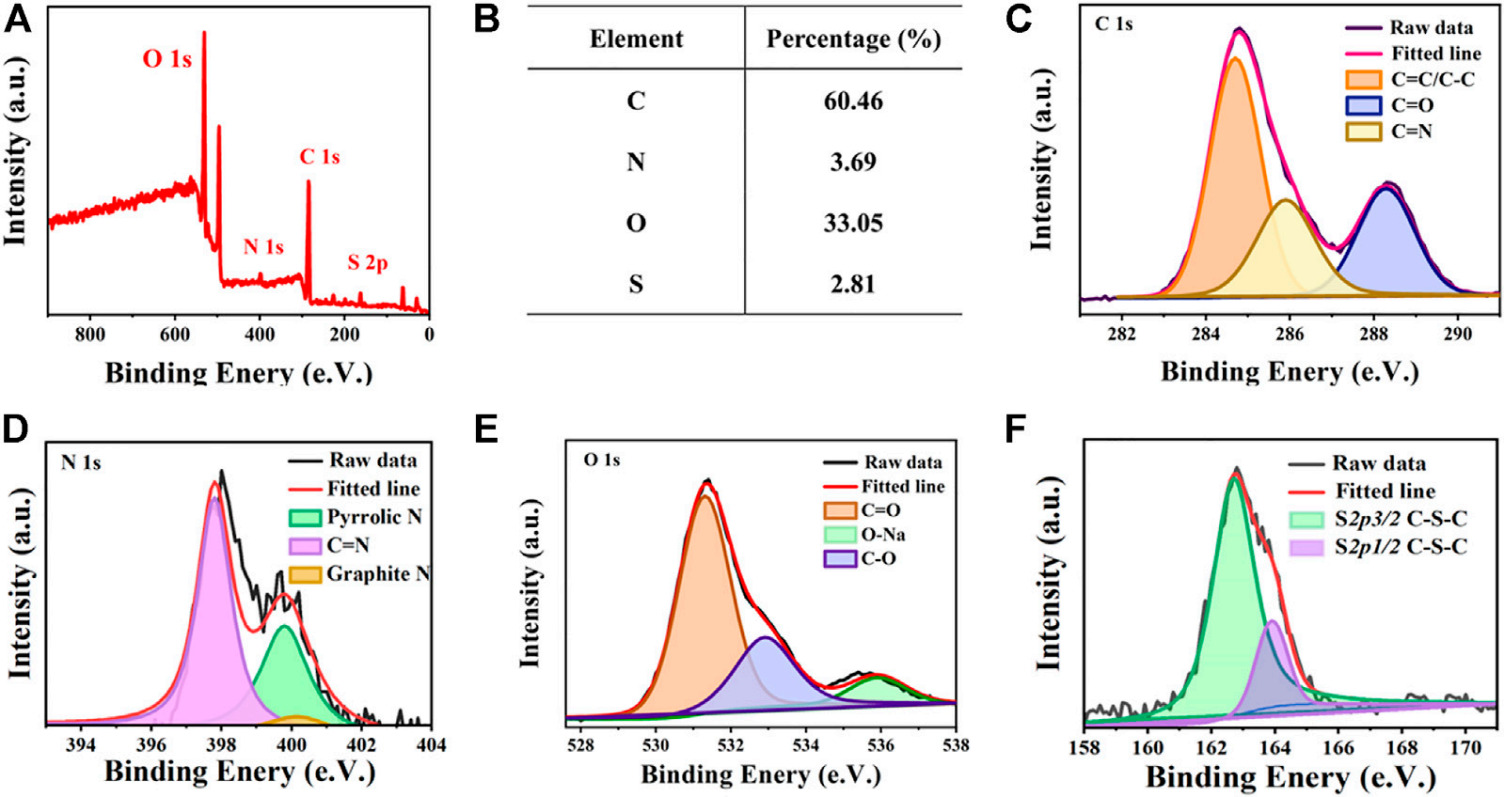
**(A)** X-ray photoelectron spectroscopy (XPS) spectrum of AIE-CDs; **(B)** proportion of each element in AIE-CDs; and high-resolution XPS spectrum of **(C)** C 1s, **(D)** N 1s, **(E)** O 1s, and **(F)** S 2p.

The chemical composition and structure of the AIE-CD surface were analyzed by X-ray photoelectron spectroscopy (XPS). As shown in Figures 3A,B, AIE-CDs are mainly composed of C 1s (284.7 eV), O 1s (531.3 eV), N1s (398.1 eV), and S 2p (162.8 eV) with percentages of 60.46%, 33.05%, 3.69%, and 2.81%, respectively. The high-resolution C 1s spectrum, shown in Figure 3C, can be divided into three peaks located at 284.7 eV, 285.8 eV, and 288.2 eV, corresponding to C=C/C-C, C–N, and C=O bonding, respectively (Wei et al., 2020). The high-resolution N 1s XPS spectrum (Figure 3D) can be fitted into three peaks of C=N (397.8 eV), pyrrolic N (399.5 eV), and graphite N (400.2 eV) (Yang et al., 2019). As shown in Figure 3E, the high-resolution of O 1s appeared at three peaks at 531.3 eV, 536.0 eV, and 532.9 eV, which were attributed to C=O, C–O, and O-Na, respectively. The O-Na groups were present since the carbon dots had been treated with NaOH to maintain stable red light (Chen et al., 2017). The S2p spectrum (Figure 3F) showed two peaks at 162.8 eV and 163.8 eV, which were corresponded to S 2p 3/2 C-S-C and S 2p 1/2 C-S-C, respectively (Miao et al., 2017). Thus, this confirms that N S element-doped AIE-CDs were successfully prepared, and polycyclic aromatic substances were formed by polymerization and carbonization.

### Constructure of test strip components

In this experiment, the strip was composed of a sample pad (20 mm × 3 mm), an NC membrane (25 mm × 3 mm), and an absorbent pad (15 mm × 3 mm). The connection parts of the components were stacked with an overlap of 2 mm, and the volume of the sample was taken as 70 uL. To improve the water absorption rate, the polyester film was soaked in a mixture of 1% PEG, 3% sucrose, 0.1% NaCl, and 0.1% Tween-20 solution at 4°C overnight and then dried at 37°C. The mouse anti-human lgM/lgG antibody (0.5 mg/ml) immobilized at the T-lines on the NC membrane with 1 μl/cm caught the target lgM/IgG against SARS-COV-2 and formed the AIE-CD-SSP–(SARS-CoV-2 IgM/IgG)–(mouse anti-human IgM/IgG) sandwich immune structure compound. Meanwhile, goat anti-chicken IgY (0.8 mg/ml) immobilized at the C-line caught AIE-CD-chicken IgY and formed AIE-CD–chicken IgY (goat anti-chicken IgY). After adding the test sample, in the presence of SARS-CoV-2 IgM/IgG, both the T-line and C-line exhibits blue and red fluorescence signals under blue and green light irradiation, while in the absence of SARS-CoV-2 IgM/IgG, only the C-line has the fluorescence signals.

### Performance of the lateral flow assay

**FIGURE 4:**
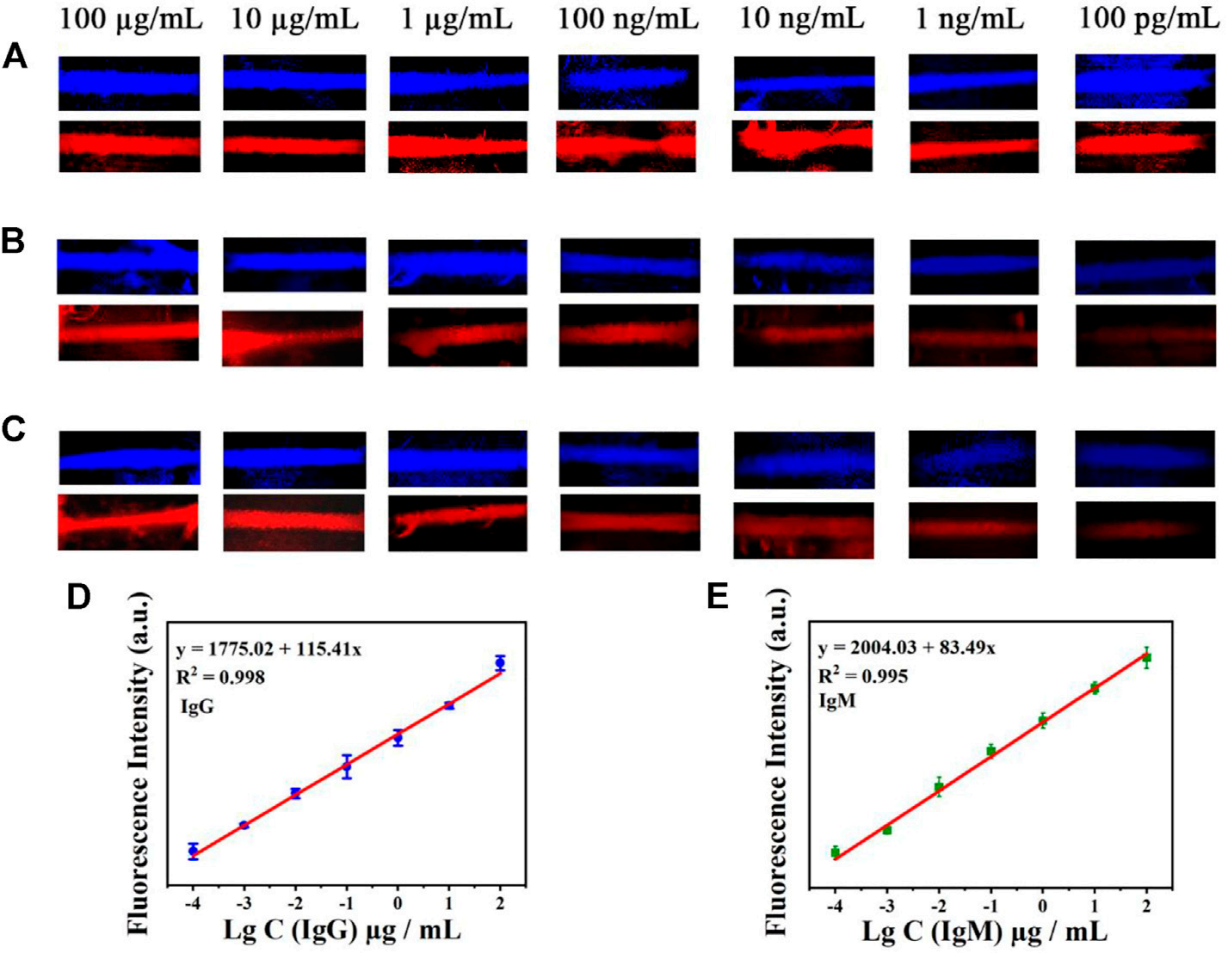
Test strip’s simultaneous detection of IgM and IgG antibodies by AIE-CD-SSP under the fluorescence microscope of blue light irradiation and green light irradiation. **(A)** C-line, **(B)** antibody IgM, and **(C)** antibody IgG (concentrations from top to bottom are 100 μg/ml, 10 μg/ml, 1 μg/ml, 100 ng/ml, 10 ng/ml, 1 ng/ml, and 100 pg/ml); the relationship between the detected fluorescence intensity of 591 nm and concentration under the excitation wavelength at 500 nm of IgG **(D)** and IgM **(E)**.

The sensitivity of the AIE-CD-SSP lateral flow immunoassay technology was investigated using different concentrations of the SARS-CoV-2 IgG and IgM solution. A series of concentrations of SARS-CoV-2 IgG and IgM solutions were added to the sample pads of the test strips. For the test, the images of the test results of the test strips under the fluorescence microscope by blue and green light irradiation are shown in Figure 4. A bright fluorescence band was observed on the C-line indicating the validity of the test. The fluorescence intensity of the T-line was positively correlated with the concentration of SARS-CoV-2 IgM and IgG, and the concentration range used was from 100 μg/ml to 100 pg/ml. As shown in Figures 4B,C, a weak fluorescent band can be observed on the T-line even when the concentration of SARS-CoV-2 antibodies was as low as 100 pg/ml, which was lower than the commercial colloidal gold detection limit of 100 ng/ml. The relationship between the detected fluorescence intensity of 591 nm of the T-line (Figures 4D,E) and concentration under the excitation wavelength at 500 nm is shown as follows: IgG *y* = 115.41 *x* + 1775.02 (*R* ^2^ = 0.998) and IgM *y* = 83.49 *x* + 2004.03 (*R* ^2^ = 0.995), which proves that the prepared LFA exhibited a highly sensitive detection of SARS-CoV-2 antibodies.

**FIGURE 5:**
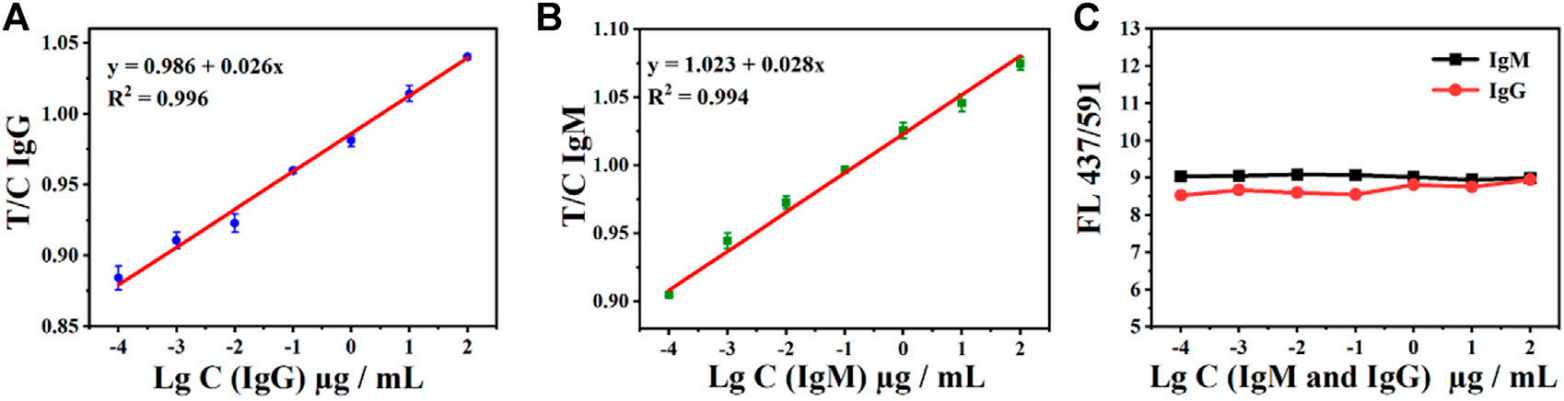
Ratio of T-line to C-line (T/C) under the excitation at 365 nm and the gradient concentration detection of **(A)** IgG and **(B)** IgM antibodies; **(C)** AIE-CD-SSP for the detection of gradient concentrations of IgM and IgG antibodies under the excitation at 365 nm and the ratio of emission fluorescence intensity at 437 nm and 591 nm FL_(437/591)_.

In addition, the relationship between the T- and C-line can also be used to detect SARS-CoV-2 IgM and IgG. The fluorescence intensity T/C of both antibody IgM and IgG exhibited a good linear relationship. As shown in Figures 5A,B, the relationship between the T/C and concentration is given as IgG *y* = 0.026 x+ 0.986 (*R* ^2^ = 0.996) and IgM *y* = 0.028 *x* +1.023 (*R* ^2^ = 0.994). In addition, the ratio of the double emission peaks of AIE-CDs also exhibited the potential to be used as an internal standard for the test. The fluorescence intensity at 437 nm and 591 nm under the excitation wavelength of 365 nm, namely, FL_437/591_, is shown in Figure 5C. The ratio (FL_437/591_) is a constant at approximately 8.5 ± 0.5 with the measurement of a series of concentrations of SARS-CoV-2 IgM and IgG (100 μg/ml to 100 pg/ml). Therefore, AIE-CD-SSP could be realized as an internal standard for the detection of IgM and IgG antibodies, which improved the detection reliability and precision.

**FIGURE 6:**
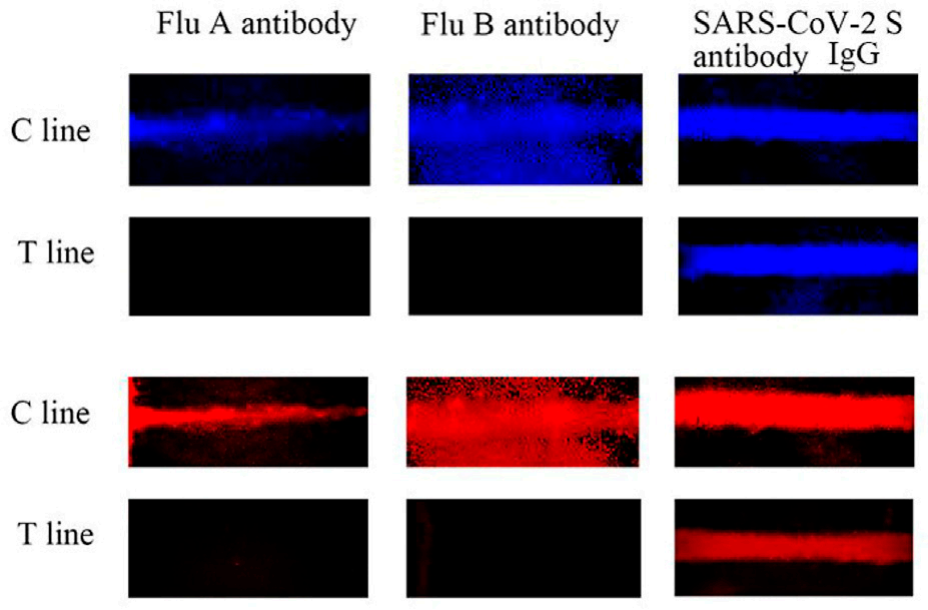
AIE-CD-SSP lateral flow immunoassay technology detecting different antibodies at 100 ng/ml using a fluorescence microscope under blue light irradiation and green light irradiation for the selective study.

To evaluate the selectivity of the AIE-CD-SSP lateral flow immunoassay system, 100 ng/ml of three different influenza virus antibodies (SARS-CoV-2 S IgG, Influenza A, and Influenza B) was tested. As shown in Figure 6, except for the specific antibody SARS-CoV-2 S IgG, no fluorescent bands were observed in the other T-lines because other influenza virus antibodies could not form a sandwich immune structure. The aforementioned results show that AIE-CD-SSP lateral flow immunoassay technology has excellent selectivity.

## Conclusion

In this study, we propose for the first time that AIE-CDs with double-emitted peaks can be used as a novel labeled luminescent material for fluorescent immunization. The fluorescent complex was formed by coupling with the SARS-CoV-2 S protein through the carbodiimide method, and the AIE-CDs were attached to the surface of the SARS-CoV-2 S protein. The successful coupling was proved by UV-vis and zeta potential characterization methods. The detection of antibodies can be achieved through the specific combination of sandwich structure “antigen–antibody–anti-antibody.” The simultaneous and separate detection of SARS-CoV-2 S IgM and IgG was achieved by fluorescent immunochromatographic test strips with a low detection limit of 100 pg/ml.

Moreover, the ratio of dual emission is a constant, which provided reliable internal standards to monitor the reliability of the sensor. The results of this work provided a new idea for the selection of fluorescent markers for fluorescent immunochromatographic test strips.

## Data Availability

The original contributions presented in the study are included in the article/Supplementary Material; further inquiries can be directed to the corresponding authors.
